# The influence of dietary and supplemental omega-3 fatty acids on the omega-3 index: A scoping review

**DOI:** 10.3389/fnut.2023.1072653

**Published:** 2023-01-19

**Authors:** Meghan Dempsey, Michelle S. Rockwell, Laurel M. Wentz

**Affiliations:** ^1^Department of Nutrition and Healthcare Management, Appalachian State University, Boone, NC, United States; ^2^Department of Human Nutrition, Foods, and Exercise, Virginia Polytechnic Institute and State University, Blacksburg, VA, United States

**Keywords:** omega-3 fatty acids, polyunsaturated fatty acid (PUFA), docosahexaenoic acid (DHA), eicosapentaenoic acid (EPA), alpha linolenic acid (ALA), omega-3 index

## Abstract

**Introduction:**

The majority of the population do not consume adequate omega-3 fatty acids (n-3 FA), leading to global deficiencies, as evidenced by poor omega-3 status. An indicator of overall n-3 FA status, omega3-index (O3i) ≥8% has been associated with reduced risk of chronic disease, most notably cardiovascular disease. Thus, a synthesis of current research summarizing the effects of n-3 FA intake on O3i is warranted to develop and refine clinical recommendations. The purpose of this scoping review was to evaluate the effect of n-3 FA interventions and estimate sufficient n-3 FA intake to improve O3i to meet recommendations.

**Methods:**

Search criteria were human studies published in English from 2004 to 2022 that assessed O3i at baseline and following an n-3 FA intervention.

**Results:**

Fifty-eight studies that met inclusion criteria were identified. Protocols included fish consumption, fortified foods, combined eicosapentaenoic acid (EPA) and docosahexaenoic acid (DHA) supplements, supplements of single n-3 FA (alpha linolenic acid (ALA), EPA, DHA, etc.), and supplements providing multiple n-3 FA. Dietary supplements varied in chemical composition; the most common were triglycerides or ethyl esters. The lowest supplementation protocol was 100 mg/d, and the largest was 4,400 mg/d EPA and DHA. Supplementation time period ranged from 3 weeks to 1 year. At baseline, three study samples had mean O3i >8%, although many intervention protocols successfully increased O3i.

**Discussion:**

Generally, the lowest doses shown to be effective in raising O3i to recommended levels were >1,000 mg/d of combination DHA plus EPA for 12 weeks or longer. Supplements composed of triglycerides were more bioavailable and thus more effective than other formulas. Based on the data evaluated, practical recommendations to improve O3i to ≥8% are consumption of 1,000–1,500 mg/d EPA plus DHA as triglycerides for at least 12 weeks.

## Introduction

Poor intake of omega-3 fatty acids (n-3 FA) is worldwide, stimulating a scientific and commercial interest in this essential lipid. High consumption of n-3 FA's has been associated with improved cardiovascular health, decreased anxiety and depression, as well as reduced rates of cancer, Alzheimer's Disease, type 1 Diabetes, multiple sclerosis, and total mortality (1). The primary dietary n-3 FA's are alpha linolenic acid (ALA; C18:3n-3), eicosapentaenoic acid (EPA; C20:5n-3), and docosahexaenoic acid (DHA; C22:6n-3). Only ALA has a dietary reference intake (DRI) of 1.1–1.6 g/d, although experts have advised recommendations for EPA and DHA (2, 3), since elongation of ALA produces limited longer chain n-3 FA (4). Sources of ALA include plant oils (e.g., flaxseed, and soybean) as well as some algae. EPA and DHA are found in fatty fish such as salmon, tuna, herring, and mackerel. Due to inadequate synthesis of EPA and DHA from ALA, many professional organizations have established recommendations for fish consumption. The 2015 Dietary Guidelines for Americans (DGA) and the American Heart Association recommend consuming two servings of fish per week (3.5–4-oz. per servings), to reach an intake of 250 mg/d of EPA and DHA. However, the majority of the American population consumes approximately half of the recommended 7–8 oz. fish per week (5). NHANES data have estimated mean fish intake among adults is 4 oz. per week and only 1 oz. per week if limiting to fish high in n-3 FA. This fish intake translates to dietary n-3 FA short of 250 mg/d, with an estimated mean consumption of 63 ± 2 mg/d DHA (72 ± 4 mg/d including supplements) and 23 ± 1 mg/d EPA (41 ± 4 mg/d including supplements). Therefore, developing evidence-based recommendations and approaches to include more n-3 FA's into the diet is essential for ensuring long term health and wellness.

Research suggests that even those who consume fish regularly have a low omega-3 index (O3i) (1), defined as the ratio of EPA and DHA to total fatty acids in erythrocyte membranes indicative of overall n-3 FA status. O3i has been shown to reflect tissue n-FA distribution and is more representative of long-term n-3 FA dietary intake than other assessment methods (6). Furthermore, O3i is commonly measured in clinical and research settings *via* quick and non-invasive blood spot analysis. However, calculation of O3i may vary by laboratory. In 2004, Harris and Schacky (7) initiated O3i research after showing that individuals with O3i >8% had lower risk of cardiovascular disease vs. those with <4%. Further evidence has supported health benefits of maintaining O3i >8% (1), which is a lofty target considering a high percentage of individuals assessed have O3i in the highest risk category <4% (8–16). Current recommendations for ALA intake and fish consumption are inadequate for most individuals to maintain a healthy O3i. According to a cross-sectional study, 83% of those consuming at least two servings of fatty fish per week had an O3i <8%, and only those consuming fish regularly plus supplementing with >1,000 mg/d EPA and DHA maintained optimal O3i (1). Globally, n-3 FA intake and blood concentrations fall well below recommendations, showing worldwide deficiency and elevated risk for chronic disease (15). Therefore, the purpose of this scoping review was to evaluate the effect of n-3 FA interventions and explore sufficient n-3 FA intake to meet O3i recommendations. While increasing dietary n-3 FA's generally improves the O3i, the response depends on type of n-3 FA, dose, chemical composition, duration, and participant characteristics (such as sex, age, genotype menopausal status among other factors that determine lipid distribution). We synthesized available literature to create practical recommendations to reach optimal O3i status in the general population.

## Methods

**Figure 1:**
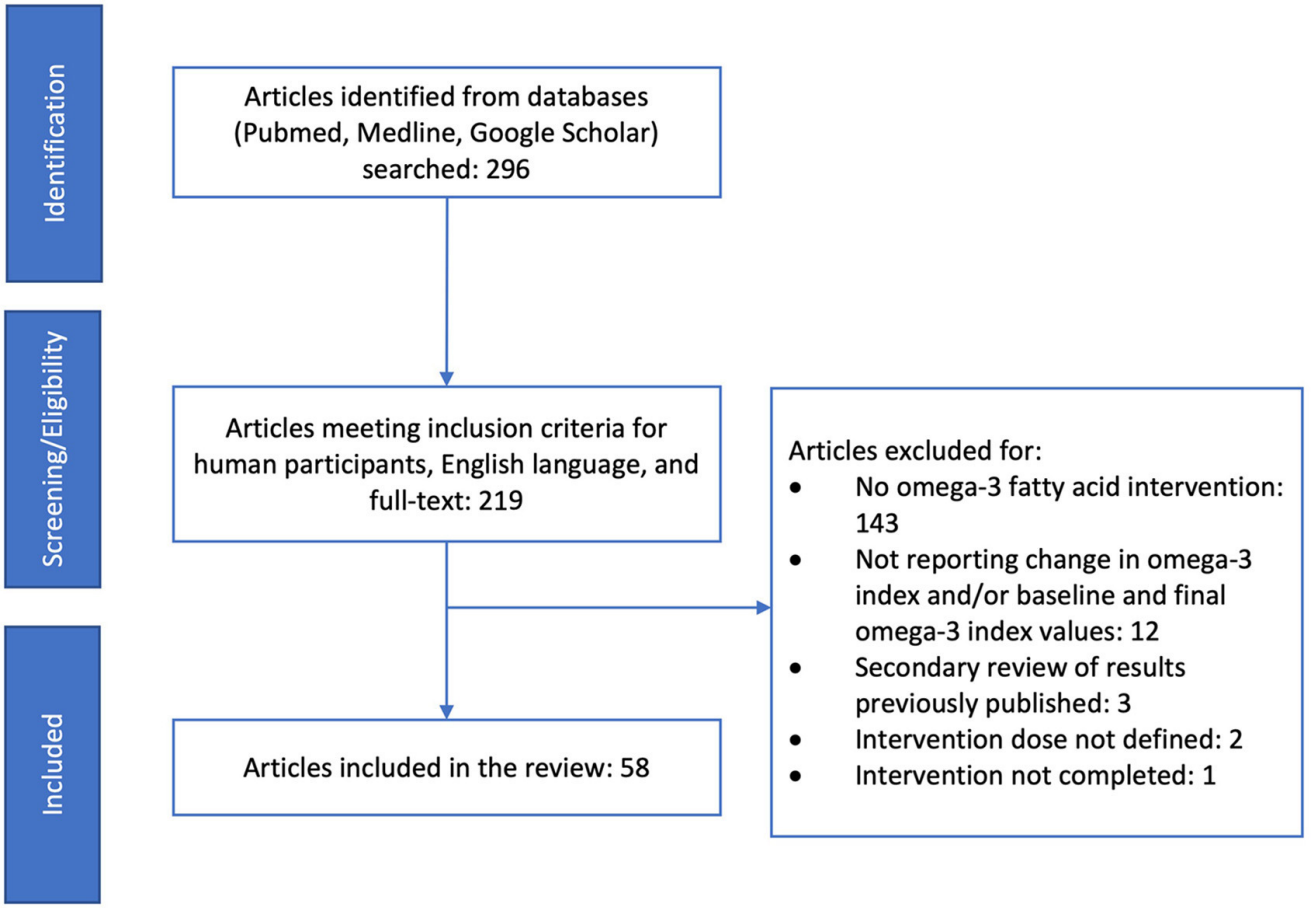
PRIMSA flow chart showing study selection for scoping review.

Research databases (Pubmed, Medline, and Google Scholar) were searched for peer-reviewed articles evaluating changes in the O3i following a dietary or supplemental intervention (Figure 1). Search dates were 2004–2022 based on the pivotal publication by Harris and Schacky that established the O3i as an indicator of cardiovascular disease risk, sparking an influx of research (7). Search terms were “omega-3 fatty acids, omega-3 fatty acid supplement, omega-3 index, ALA, EPA, DHA.” Inclusion criteria were publication in English, human participants, defined n-3 FA intervention, and assessment of O3i at baseline as well as follow-up. Studies were excluded for including previously published intervention data or using retrospective data. To be evaluated, studies were required to specify n-3 FA acid intervention by dose and time period as well as present baseline and follow-up O3i. Types of interventions include dietary supplements, fortified foods, oils, and fish containing EPA, DHA, ALA, or a combination of mixed n-3 FA. There were no limitations based on participant age, health status, pregnancy, or other conditions.

## Results

### Search results

**Figure 2:**
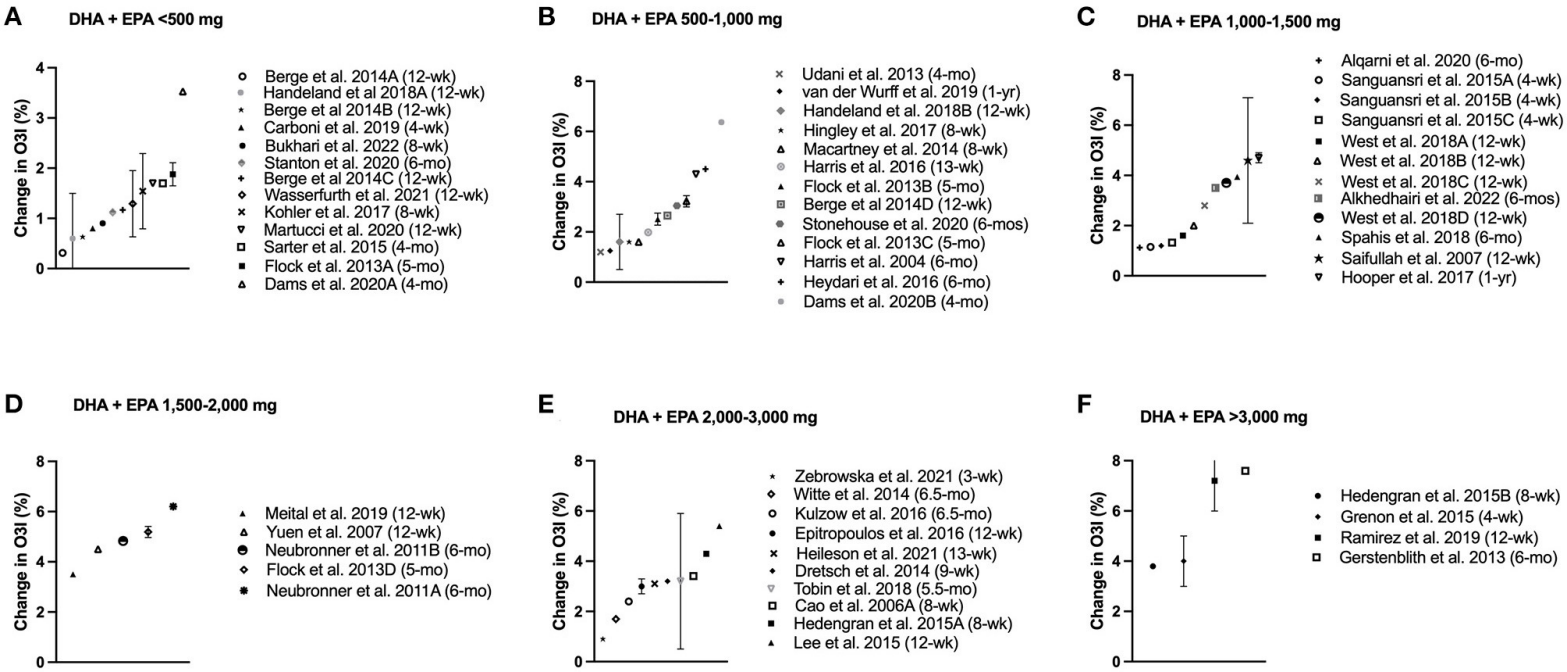
Change in omega-3 index (O3i) from baseline for protocols using combined EPA + DHA separated by dose. Data are presented as means ± SD (if available in publication). Legend shows length of supplementation/fortification in parentheses. **(A)** Doses less than 500 mg/d; **(B)** Doses ranging from 500–1,000 mg/d; **(C)** Doses ranging from 1,000–1,500 mg/d; **(D)** Doses ranging from 1,500–2,000 mg/d; **(E)** Doses ranging from 2,000–3,000 mg/d; **(F)** Doses exceeding 3,000 mg/d.

We identified 58 original research articles that evaluated the effect of n-3 FA on O3i (Tables 1–4; Figures 2–4). Many studies used multiple interventions to compare formulas, doses, and type of n-3 FA; thus, the total number of individual cohorts presented exceeds 58 articles. These intervention protocols included dietary consumption of fish (*n* = 1), fortified foods (*n* = 9), oils (*n* = 6), supplements of single n-3 FA (*n* = 16), and supplements providing a combination n-3 FA (*n* = 52). Dietary supplements varied in chemical composition; the most common were triglycerides (TAG) or ethyl esters (EE). The lowest intervention dose was supplemental 100 mg/d EPA plus DHA (8), and the largest was 4,400 mg/d EPA plus DHA (17, 18). Time period of intervention ranged from 3 weeks (19) to 1 year (16, 20).

**Table 1:** Combination EPA plus DHA supplementation protocols and omega-3 index (O3i).

**References**	**Supplementation protocol**	**Participants**	**Baseline O3i (%)**	**Final O3i (%)**
Saifullah et al. (12)	680 mg EPA + 340 mg/d DHA for 12 wks	*N* = 15 adults (73% male) mean age 58 ± 12 yrs receiving hemodialysis (USA)	2.1 ± 1.1	6.8 ± 2.6^*^
Bukhari et al. (26)	Fortified food: Estimated 115 (±68) mg EPA + 246 (±129) mg/d DHA for 8 wks	*N* = 38 military personnel (79% male) mean age 24 ± 10 yrs (USA)	2.8 ± 0.7	3.7 ± 0.7^*^
Alqarni et al. (54)	840 mg EPA + 560 mg/d DHA for 6 mos	*N* = 114 individuals (52% male) median age 17 yrs at ultra-high risk of developing psychosis (International)	Median: 3.0	Median: 4.1^*^
Spahis et al. (14)	1,200 mg/d EPA + DHA for 6 mos	*N* = 11 male children aged 8–18 yrs with severe non-alcoholic fatty liver disease (French Canada)	3.0 ± 0.2	6.9 ± 0.4^*^
Sarter et al. (13)	82 mg EPA + 172 mg/d DHA for 4 mos	*N* = 46 adult vegans aged >20 yrs with O3i <4% (USA)	3.1 ± 0.6	4.8 ± 0.8^*^
Dretsch et al. (9)	1,175 mg EPA + 950 mg/d DHA (EE) for 9 wks	*N* = 44 soldiers (93% male) mean age 31 ± 7 yrs on deployment (USA)	3.5 ± 7.1	6.7 ± 1.7^*^
Berge et al. (8) A	67 mg EPA + 33 mg/d DHA for 12 wks	*N* = 53 adults (66% male) mean age 46 ± 13 yrs with high triglycerides (Canada)	3.7 ± 0.9	4.0 ± 0.8^*^
Berge et al. (8) B	134 mg EPA + 66 mg/d DHA for 12 wks	*N* = 53 adults (64% male) mean age 41 ± 13 yrs with high triglycerides (Canada)	3.6 ± 0.8	4.2 ± 0.8^*^
Berge et al. (8) C	268 mg EPA + 132 mg/d DHA for 12 wks	*N* = 51 adults (73% male) mean age 44 ± 12 yrs with high triglycerides (Canada)	4.0 ± 0.9	5.2 ± 1.0^*^
Berge et al. (8) D	536 mg EPA + 264 mg/d DHA for 12 wks	*N* = 58 adults (67% male) mean age 46 ± 12 yrs with high triglycerides (Canada)	3.7 ± 0.7	6.3 ± 1.0^*^
Van der Wurff et al. (16)	520 mg EPA + 280 mg/d DHA for 1 yr	*N* = 108 adolescents (57% male) mean age 14 ± 1 yrs with an O3i ≤5% (The Netherlands)	3.7 ± 0.5	4.9 ± 1.4^*^
Zebrowska et al. (19)	852 mg EPA + 1,602 mg/day DHA for 3 wks	*N* = 12 non-elite male endurance runners mean age 33 ± 7 yrs (Poland)	3.9 ± 0.5	4.8 ± 0.8^*^
Hooper et al. (20)	225 mg EPA + 800 mg/d DHA for 1 yr	*N* = 98 elderly adults (69% female) mean age 80 ± 5 yrs with memory loss (France)	4.1 ± 0.6	8.8^*^^#^
Köhler et al. (28)	Fortified sausages providing ~250 mg/d EPA + DHA (EE) for 8 wks	*N* = 22 healthy adults (59% male) mean age 26 ± 8 yrs (Germany)	4.2 ± 0.5	5.7 ± 0.7^*^
Epitropoulos et al. (55)	1,680 mg EPA + 560 mg/d DHA (re-esterified EE) for 12 wks	*N* = 54 adults (70% female) mean age 57 ± 17 yrs with dry eye disease (USA)	4.2 ± 1.0	7.2 ± 2.7^*^
Carboni et al. (27)	Fortified mussels providing ~300 mg/d EPA + DHA for 4 wks	*N* = 12 university staff and students (67% male) mean age 24 ± 1 yrs (UK)	4.3 ± 0.8	5.1 ± 1.0^*^
Heileson et al. (56)	560 mg EPA + 2,000 mg DHA + 320 mg/d DPA for 13 wks	*N* = 31 American male football players aged >18 yrs (USA)	4.3 ± 0.9	7.4^*^^#^
Flock et al. (50) A	191 mg EPA + 121 mg/d DHA for 5 mos	*N* = 23 healthy adults (55% male) mean age 26 ± 2 yrs (USA)	4.3 ± 0.2	6.2 ± 0.2^*^
Flock et al. (50) B	374 mg EPA + 237 mg/d DHA for 5 mos	*N* = 22 healthy adults (52% female) mean age 27 ± 2 yrs (USA)	4.3 ± 0.2	6.8 ± 0.2^*^
Flock et al. (50) C	556 mg EPA + 352 mg/d DHA for 5 mos	*N* = 24 healthy adults (54% male) mean age 26 ± 1 yrs (USA)	4.3 ± 0.3	7.5 ± 0.2^*^
Flock et al. (50) D	1,103 mg EPA + 698 mg/d DHA for 5 mos	*N* = 24 healthy adults (54% male) mean age 26 ± 1 yrs (USA)	4.3 ± 0.2	9.5 ± 0.2^*^
Cao et al. (57) A	1,296 mg EPA + 864 mg/d DHA for 8 wks	*N* = 9 healthy adults (60% female) mean age 49 ± 8 yrs (USA)	4.3 (SE 0.4)	7.8 (SE 0.3)^*^
Stanton et al. (30)	Fortified chicken and eggs providing ~150 mg/d EPA + DHA for 6 mos	*N* = 33 healthy adults (59% female) mean age 41 ± 11 yrs (Ireland)	4.3 ± 1.6	5.4^*^^#^
Meital et al. (58)	300 mg EPA + 1,500 mg/d DHA for 12 wks	*N* = 15 elderly male patients mean age 74 ± 5 yrs with abdominal aortic aneurysm (Australia)	4.5 ± 0.2	8.0 ± 0.2^*^
Sanguansri et al. (35) A	618 mg EPA + 395 mg/d DHA for 4 wks (milk protein-sugar)	*N* = 14 healthy adults (64% female) mean age 58 (SE 3) yrs (Australia)	4.7 (SE 0.3)	5.8 (SE 0.3)^*^
Sanguansri et al. (35) B	629 mg EPA + 397 mg/d DHA for 4 wks (milk protein-sugar-resistant starch)	*N* = 16 healthy adults (63% female) mean age 56 (SE 2) yrs (Australia)	5.1 (SE 0.2)	6.3 (SE 0.2)^*^
Sanguansri et al. (35) C	647 mg EPA + 402 mg/d DHA for 4 wks (capsules consumed with milk)	*N* = 17 healthy adults (71% female) mean age 56 (SE 3) yrs (Australia)	4.5 (SE 0.1)	5.8 (SE 0.2)^*^
Macartney et al. (59)	140 mg EPA + 560 mg/d DHA for 8 wks	*N* = 13 healthy adult males mean age 24 (SE 7) yrs (Australia)	4.7 (SE 0.2)	6.3 (SE 0.3)^*^
Hingley et al. (60)	140 mg EPA + 560 mg/d DHA (TAG) for 8 wks	*N* = 13 trained male cyclists mean age 24 ± 7 yrs (Australia)	4.7 ± 0.2	6.3 ± 0.3^*^
Harris et al. (47)	1,000 mg/d EPA + DHA (TAG) for 6 mos	*N* = 21 adult (64% male) mean age 55 ± 9 yrs recipients of a heart transplant (Canada)	4.7 ± 1.1	9.0 ± 1.7^*^
Harris et al. (61)	1,000 mg/d n-3 FA (EE; including 850 mg EPA + DHA) for 13 wks	*N* = 230 adults (77% male) mean age 67 ± 11 yrs with chronic heart failure (Italy)	4.8 ± 1.7	6.7 ± 1.9^*^
Tobin et al. (33)	1,380 mg EPA and 1,140 mg/d DHA (EE) for 5.5 mos	*N* = 81 patients (56% female) mean age 55 ± 13 yrs with non-alcoholic fatty liver disease (USA)	4.8 ± 1.1	8.0 ± 2.6^*^
Yuen et al. (62)	1,000 mg EPA + 700 mg/d DHA for 12 wks	*N* = 29 epileptic patients (UK); sex and age not provided	4.8 ± 1.6	9.3^*^
Fischer et al. (34)^**×**^	Weeks 1–4: 460 mg EPA + 380 mg/d DHA (EE) Weeks 5–8: 980 mg EPA + 760 mg/d DHA (EE)	*N* = 20 healthy adults with O3i <6% (Germany) 50% male mean age 32 ± 8 yrs 50% female mean age 38 ± 6 yrs	4.9 ± 0.2	8.4 ± 0.2^*^
Gerstenblith et al. (32)	840 mg EPA + 2,520 mg/d DHA (TAG) for 6 mos	*N* = 17 older adults (71% female) mean age 69 yrs with macular degeneration (USA)	5.0	12.6^*^^#^
West et al. (63) A	684 mg EPA + 549 mg/d DHA (EE) for 12 wks	*N* = 19 healthy adults (53% female) mean age 41 ± 14 yrs (UK)	4.8 ± 0.8	6.4 ± 0.9^*^
West et al. (63) B	381 mg EPA + 888 mg/d DHA (EE) for 12 wks	*N* = 20 healthy adults (50% female) mean age 39 ± 13 yrs (UK)	5.2 ± 0.9	7.2 ± 1.0^*^
West et al. (63) C	726 mg EPA + 576 mg/d DHA in a self-micro-emulsifying delivery system for 12 wks	*N* = 19 healthy adults (53% male) mean age 40 ± 13 yrs (UK)	5.1 ± 0.9	7.9 ± 0.9^*^
West et al. (63) D	408 mg EPA + 918 mg/d DHA in a self-micro-emulsifying delivery system for 12 wks	*N* = 20 healthy adults (50% female) mean age 39 ± 13 yrs (UK)	5.3 ± 1.1	9.0 ± 1.2^*^
Grenon et al. (17)	2,600 mg EPA + 1,800 mg/d DHA for 4 wks	*N* = 36 older adults (98% male) mean age 68 ± 7 yrs with peripheral artery disease (USA)	5.2 ± 1.7	9.2^*^^#^
Heydari et al. (64)	465 mg EPA + 375 mg/d DHA (EE) for 6 mos	*N* = 180 older adults (82% male) mean age 60 ± 10 yrs with acute myocardial infarction (USA)	5.5 ± 1.8	10^*^^#^
Ramirez et al. (18)	2,600 mg EPA + 1,800 mg/d DHA for 12 wks	*N* = 11 older male adults mean age 69 ± 8 yrs with peripheral artery disease (USA)	5.5 ± 2.1	12.7^*^^#^
Martucci et al. (29)	Fortified milk providing 350 mg/d EPA + DHA for 12 wks	*N* = 48 community-dwelling elderly adults (54% male) mean age of 70 ± 5 yrs (Italy)	5.6	7.3^*^
Handeland et al. (31) A	Fish providing 150 mg EPA + 257 mg/d DHA for 12 wks	*N* = 128 adolescents (56% female) mean age 15 ± 0.3 yrs (Norway)	5.8 ± 1.2	6.4 ± 1.2^*^
Handeland et al. (31) B	463 mg EPA + 319 mg/d DHA for 12 wks	*N* = 142 adolescents (54% female) mean age 15 ± 0.3 yrs (Norway)	5.7 ± 1.3	7.3 ± 1.2^*^
Stonehouse et al. (37)	600 mg EPA + 280 mg/d DHA for 6 mos	*N* = 106 healthy adults (56% female) mean age 56 ± 7 yrs with knee osteoarthritis (Australia)	6.0 ± 1.3	9.0 ± 1.6^*^
Wasserfurth et al. (65)	109 mg EPA + 87 mg DHA + 124 mg/d SDA for 12 weeks	*N* = 33 older adults (69% female) mean age 59 ± 6 yrs (Germany)	6.1 ± 1.3	7.4 ± 1.1^*^
Udani and Ritz (66)	756 mg EPA + 228 mg/d DHA for 4 mos	*N* = 157 healthy adults (sex not specified) mean age 44 ± 1 yrs (USA)	6.1	7.3^*^
Alkhedhairi et al. (53)	772 mg EPA + 384 mg/d DHA for 6 mos	*N* = 49 inactive older adults (53% female) mean age 71 ± 5 yrs (UK)	6.5 ± 1.7	10.0 ± 2.1^*^
Hedengran et al. (46) A	767 mg EPA + 1,930 mg/d DHA (acyl glycerol) for 8 wks	*N* = 36 adults (92% male) mean age 63 ± 8 yrs with high fasting triglycerides (USA)	6.8 ± 1.9	11.1^*^
Hedengran et al. (46) B	1,702 mg EPA + 1,382 mg/d DHA (EE) for 8 wks	*N* = 36 adults (65% male) mean age 60 ± 11 yrs with high fasting triglycerides (USA)	6.5 ± 1.5	10.3^*^
Neubronner et al. (36) A	1,010 mg EPA + 670 mg/d DHA (rTAG) for 6 mos	*N* = 41 dyslipidemic adults (56% male) mean age 61 ± 10 yrs (Germany)	7.0 ± 1.9	13.3 ± 2.4^*^
Neubronner et al. (36) B	1,010 mg EPA + 670 mg/d DHA (EE) for 6 mos	*N* = 45 dyslipidemic adults (56% male) mean age 60 ± 9 yrs (Germany)	7.4 ± 1.8	12.2 ± 2.3^*^
Külzow et al. (67)	1,320 mg EPA + 880 mg/d DHA for 6.5 mos	*N* = 22 healthy older adults (55% male) mean age 63 ± 6 yrs (Germany)	7.8 ± 2.6	10.2 ± 2.9^*^
Lee et al. (68)	1,104 mg EPA + 912 mg/d DHA for 12 wks	*N* = 8 adults (75% female) mean age 60 ± 7 yrs on hemodialysis (South Korea)	7.8 ± 3.4	13.2 ± 2.2^*^
Witte et al. (24)	1,320 mg EPA + 880 mg/d DHA for 6.5 mos	*N* = 33 healthy older adults (53% male) mean age 65 ± 6 yrs (Germany)	8.0 ± 2.5	9.7 ± 2.9^*^
Dams et al. (23) A	190 mg EPA + 225 mg/d DHA for 4 mos	*N* = 17 healthy adults (53% male) mean age 43 ± 12 yrs with BMI 20–35 kg/m^2^ (Austria)	8.2 ± 2.3	11.7 ± 2.5^*^
Dams et al. (23) B	380 mg EPA + 450 mg/d DHA for 4 mos	*N* = 18 healthy adults (50% male) mean age 42 ± 13 yrs with BMI 20-35 kg/m^2^ (Austria)	8.1 ± 2.6	14.4 ± 2.7^*^

Research studies compared different doses, forms of n-3 FA, or composition; thus, each intervention protocol has a separate entry. O3i are presented as means ± SD unless otherwise noted.

* p < 0.05;

# calculated final O3i;

× excluded from Figure 2 due to inconsistent dosage. TAG, triglycerides; rTAG, re-esterified triglycerides; EE, ethyl esters; DPA, docosapentaenoic acid; SDA, stearidonic acid.

Study participants resided in a variety of countries and continents, including North America, Europe, Asia, and Australia. Characteristics of individuals varied widely, showing strong representation of age ranges, male and female, and healthy as well as those with chronic disease. In this analysis, sex, age, and chronic disease appeared to have no effect on the response to n-3 FA supplementation compared to healthy individuals. The lowest mean baseline O3i was 1.1 ± 1.2% in Italian children with hyperlipidemia (21), and the highest was 9.4 ± 3.7% in healthy German adults (22). Fatty acid extraction and calculation of O3i differed between these laboratories, thus making direct comparison of O3i concentrations challenging.

Three studies (5 sample cohorts) reported mean baseline O3i ≥8%, which were measured in Austrian and German laboratories (22–24). About half of the studies reviewed found that participants' O3i increased to ≥8% following the intervention period, although nearly all supplementation protocol significantly improved O3i from baseline. The smallest dose to reach >8% O3i was 200 mg/day of DHA for nearly 6 months in pregnant women (25) (Table 3, 1.5% increase from baseline). The largest dose, used in two studies, was 4,400 mg/day combination of DHA and EPA for 1–3 months (17, 18) (Table 1, 4–7% increase from baseline). The largest overall improvement in O3i was 9% increase from baseline following 6 weeks of fortified margarine containing 2,300 mg/d DHA (22) (Table 3). While fish consumption and other food fortification protocols significantly increased O3i from baseline, the magnitude of change was smaller compared to supplemental n-3 FA. Consumption of oils had little impact on O3i, showing mixed statistical changes with minimal clinical significance (Table 4). In general, participants' O3i levels increased by a greater magnitude as supplementation dose increased (Figures 2–4).

### DHA and EPA

Fifty-eight intervention protocols (from 43 publications) used a combination of EPA and DHA (Table 1). Participants consumed supplemental dosages ranging from 100 (8) to 4,400 mg/d (17, 18) over study periods from as little as 3 weeks (19) to as long as 1 year (16, 20). Food fortification protocols provided considerably lower EPA and DHA compared to supplements, ranging from an estimated 150 mg/d to 350 mg/d (26–30). Dietary n-3 FA from fish consumption had poor study compliance with fewer than half of participants willing to consume fish three time per week, thus falling short of target 400 mg/d (31).

All combination EPA/DHA interventions show statistically significant improvement in O3i, with magnitude of change ranging from 0.3 (8) to 7.6% (32) (Figure 2). Twenty of these intervention protocols (34%) improved participant O3i ≥8%, plus three that significantly increased O3i despite baseline levels ≥8% (23, 24). The most conservative dose to raise O3i ≥8% was 840 mg/d EPA and DHA ethyl esters for 6 months in American adults with non-alcoholic fatty liver disease, whose O3i rose from 5 to 8% (33). Four months of a smaller dose (415 mg/d) mixed with fruit and vegetable juice showed similar 3% improvement in Austrian participants whose baseline O3i exceeded 8% (23). While this supplementation period exceeded many protocols, other research has demonstrated that conservative doses were effective over a shorter supplementation period. In a sample of German adults, participants consumed 840 mg/d EPA and DHA for the first 4 weeks, followed by 1,680 mg/d EPA and DHA for the next 4 weeks to raise O3i from 5% to >8% (34). It remains unclear whether the initial dose would have achieved O3i within the recommended range, as levels had increased significantly above baseline before the dose was increased. The study that achieved the largest overall increase in O3i (5–13%) supplemented 3,360 mg/d EPA plus DHA for 6 months, improving baseline levels by nearly 8% in a small sample of older American adults (32).

No protocol supplementing EPA and DHA improved mean baseline O3i from <4% to ≥8%, suggesting that high doses over longer time periods are required to bridge the gap between population's O3i levels and optimal recommendations. In general, supplementation with 1,000–1,500 mg EPA and DHA raised O3i by 2–5% regardless of baseline status (Figure 2C). French adults improved from 4 to 9% by supplementing with 1,025 mg/day EPA and DHA for 1 year, showing evidence of the lowest baseline O3i levels that improved to ≥8% (20). American adults receiving dialysis starting with lower O3i had a similar improvement (2–7%), with 1,020 mg/d EPA and DHA for 3 months (12). The shortest protocol to elicit a large change in O3i supplemented 4,400 mg/d EPA and DHA for 1 month, raising O3i from 5 to 9% in American adults (17). Among the highest doses published, this study shows short-term, high-potency supplementation protocols have a comparable O3i improvement to 1,000 mg/d n-3 FA for 3 months or more. Other 3–4 week interventions had ~1% increase in O3i at lower doses, ranging from 300 mg/d in fortified mushrooms to 2,454 mg/d from supplements (19, 27, 35). Most intervention periods were 3–5 months, with many longer interventions showing plateau in O3i following 3 months of supplementation (33, 36, 37).

### EPA

Eight intervention protocols supplemented solely with EPA, half of which induced ~ 3% increase in O3i (Table 2, Figure 3). Supplementation with 2,700 mg/d of EPA in re-esterified triglycerides (rTAG) for 10 weeks in Canadian adults increased baseline O3i from 6 to 9% (38). Supplemental doses >3,000 mg/d EPA resulted in a similar 3% O3i increase in two different samples of Canadian adults over 12 weeks, but baseline levels started <4% (10, 39). Likewise, O3i in German adults increased by 3% following 6 weeks of 2,200 mg/d EPA consumed in fortified margarine, but participants had high starting values and O3i showed a high degree of variation (9.4 ± 3.5 to 12.9 ± 3.4%) (22). Lower doses did not have a clinically significant effect on O3i, as 1,000–1,500 mg/d EPA supplementation for 12–16 weeks raised baseline levels by ~ 1% (40–42).

**Table 2:** Eicosapentaenoic acid (EPA) supplementation protocols and omega-3 index (O3i).

**References**	**Supplementation protocol**	**Participants**	**Baseline O3i (%)**	**Final O3i (%)**
Klingel et al. (39) A	3,000 mg/d EPA (TAG) for 12 wks	*N* = 29 young, healthy adults (52% male) mean age 21 ± 2 yrs (Canada)	3.5 ± 0.1	6.5 ± 0.2^*^
Lee et al. (10) A	3,252 mg/d EPA for 12 wks	*N* = 28 young, healthy adults (50% male) mean age 21 ± 2 yrs (Canada)	3.6 ± 0.1	6.5^*^^#^
Harris et al. (40) A	980 mg EPA + 1,700 mg/d ALA (EE) for 4 mos	*N* = 11 healthy adults (64% female) mean age 48 ± 10 yrs with O3i ≤5% (USA)	4.1 ± 0.9	5.1 ± 0.8^*^
Lemke et al. (42) A	1,000 mg EPA + 1,000 mg/d ALA for 12 wks	*N* = 62 healthy adults (57% female) mean age 48 ± 11 yrs (USA)	4.3 ± 1.2	4.9 ± 0.1^*^
Lemke et al. (41) A	1,500 mg/d EPA (EE) for 12 wks	*N* = 26 healthy adults (65% female) mean age 49 (SE 2) yrs (USA)	4.4 (SE 0.3)	5.8 (SE 0.3)^*^
Rao et al. (69)	250 mg/d EPA for 12 wks	*N* = 53 healthy adults (59% female) mean age 54 ± 11 yrs (Australia)	5.0 ± 0.9	5.8 ± 0.9^*^
Allaire et al. (38) A	2,700 mg/d EPA (rTAG) for 10 wks	*N* = 121 adults (69% female) with abdominal obesity and elevated CRP (Canada) Female mean age 50 ± 16 yrs Male mean age 57 ± 12 yrs	6.2 ± 0.1	9.5 ± 0.1^*^
Egert et al. (22) A	Fortified margarine providing 2,200 mg/d EPA for 6 wks	*N* = 25 healthy adults (64% female) mean age 25 ± 4 yrs (Germany)	9.4 ± 3.5	12.9 ± 3.4^*^

Research studies compared different doses, forms of n-3 FA, or composition; thus, each intervention protocol has a separate entry. O3i are presented as means ± SD unless otherwise noted.

* p < 0.05;

# calculated final O3i. ALA, alpha-linolenic acid; TAG, triglycerides; rTAG, re-esterified triglycerides; EE, ethyl esters; CRP, C-reactive protein.

**Figure 3:**
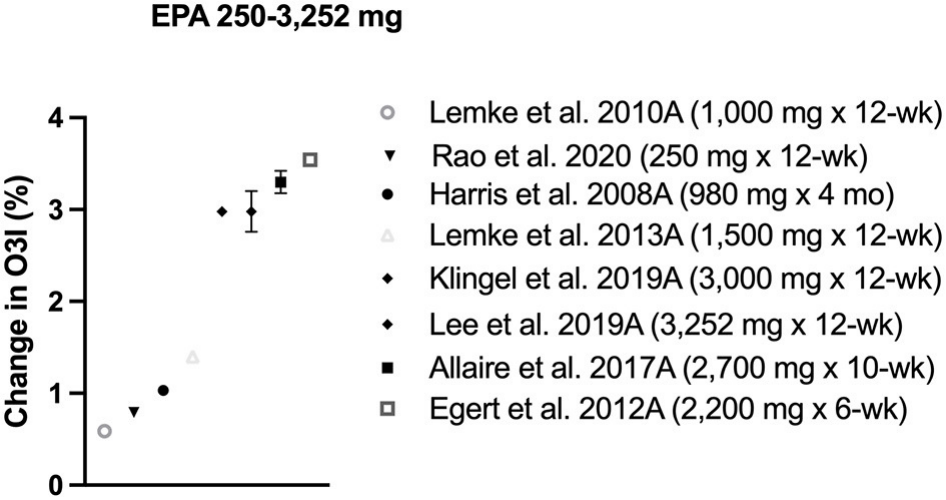
Change in omega-3 index (O3i) from baseline for protocols using EPA. Data are presented as means ± SD (if available in publication). Legend shows dose and length of supplementation/fortification in parentheses.

### DHA

Most supplementation with DHA was linked to 4–5% increase in O3i, regardless of baseline values (Table 3, Figure 4). Of the seven intervention protocols that supplemented with DHA, all participants' final mean O3i exceeded 8%, including two samples with baseline O3i <4% (Table 3, Figure 4). Two groups of Canadian adults consuming >3,000 mg/d DHA for 12 weeks improved O3i from 3.5 to 8.4% (10, 39). Surprisingly, this 5% improvement was exceeded in German adults, whose O3i rose from 9.4 ± 3.7 to 18.5 ± 4.2% with consumption of 2,300 mg/d DHA in a fortified margarine for 6 weeks (22). The most conservative dose to improve O3i >8% supplemented 200 mg/day DHA for ~ 5 months in Italian pregnant women, although their baseline O3i was 6.5 ± 1.4% so the magnitude of increase was smaller than protocols using a larger dose (25). Taken together, the evidence suggests that supplementing with DHA raised O3i to a greater extent than EPA or combination formulas.

**Table 3:** Docosahexanoic acid (DHA) supplementation protocols and omega-3 index (O3i).

**References**	**Supplementation protocol**	**Participants**	**Baseline O3i (%)**	**Final O3i (%)**
Klingel et al. (39) B	3,000 mg/day DHA (TAG) for 12 wks	*N* = 30 young, healthy adults (50% male) mean age 22 ± 2 yrs (Canada)	3.5 ± 0.1	8.4 ± 0.2^*^
Lee et al. (10) B	3,256 mg/d DHA for 12 wks	*N* = 28 young, healthy adults (50% male) mean age 22 ± 2 yrs (Canada)	3.6 ± 0.1	8.4^*^^#^
Mazahery et al. (44)	722 mg/d DHA for 1 yr	*N* = 23 children (78% male) mean age 5 ± 2 yrs with autism spectrum disorder (New Zealand)	Median: 4.7	Median: 9.1^*^^#^
Geppert et al. (45)	940 mg/d DHA for 8 wks	*N* = 52 healthy adult (76% female) mean age 26 ± 6 yrs vegetarians (Germany)	4.8 ± 0.2	8.4 ± 0.2^*^
Allaire et al. (38) B	2,700 mg/d DHA (rTAG) for 10 wks	*N* = 123 adults (69% female) with abdominal obesity and elevated CRP (Canada) Female mean age 50 ± 16 yrs Male mean age 57 ± 12 yrs	6.2 ± 0.1	11.7 ± 0.1^*^
Massari et al. (25)	200 mg/d DHA for ~ 5 mos	*N* = 65 pregnant, healthy females mean age 31 ± 5 yrs (Italy)	6.5 ± 1.4	8.0 ± 1.6^*^
Egert et al. (22) B	Fortified margarine providing 2,300 mg/d DHA for 6 wks	*N* = 25 healthy adults (60% female) mean age 25 ± 3 yrs (Germany)	9.4 ± 3.7	18.5 ± 4.2^*^

Research studies compared different doses, forms of n-3 FA, or composition; thus, each intervention protocol has a separate entry. O3i are presented as means ± SD unless otherwise noted.

* p < 0.05;

# calculated final O3i. TAG, triglycerides; rTAG, re-esterified triglycerides.

**Figure 4:**
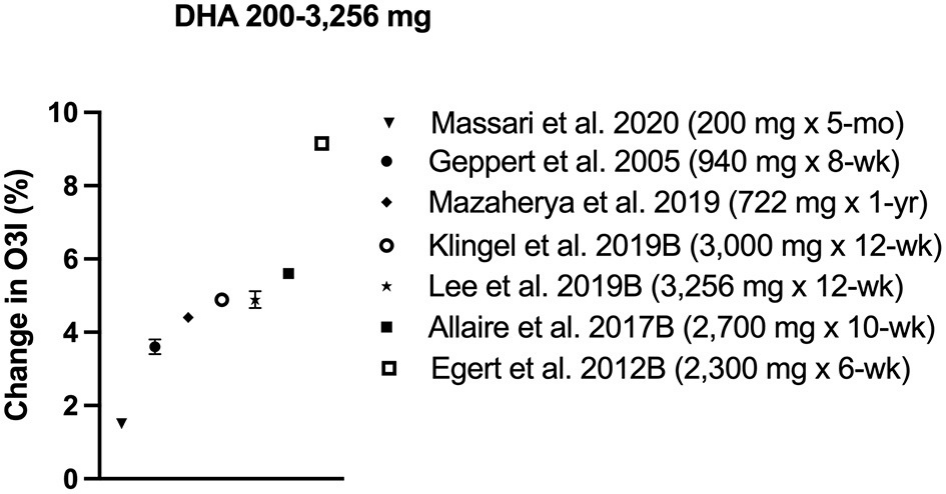
Change in omega-3 index (O3i) from baseline for protocols using DHA. Data are presented as means ± SD (if available in publication). Legend shows dose and length of supplementation/fortification in parentheses.

### Other omega-3 forms

No other form of n-3 FA had a clinically significant effect on the O3i (Table 4). While some interventions showed a small statistically significant increase in O3i, no protocol raised O3i more than about 1%, and in some cases O3i decreased from baseline. Sources of n-3 FA were flaxseed, rapeseed, sunflower, and hempseed oils high in ALA or soybean and echium oils high in stearidonic acid (SDA; C18:4n-3). Because ALA and SDA have limited elongation to EPA and DHA, consumption of these shorter chain n-3 FA have little effect on the O3i.

**Table 4:** Other omega-3 fatty acid (n-3 FA) supplementation protocols and omega-3 index (O3i).

**References**	**Supplementation protocol**	**Participants**	**Baseline O3i (%)**	**Final O3i (%)**
Del Bo' et al. (21)	Hempseed oil providing 700 mg ALA + 1,400 mg/d LA for 8 wks	*N* = 18 children and adolescents (64% male) mean age 12 ± 2 yrs with primary hyperlipidemia (Italy)	1.1 ± 1.2	2.3^*^^#^
Pieters and Mensink (11)	Echium oil providing 1,200 mg/d SDA for 6 wks	*N* = 32 overweight adults (50% male) mean age 51 ± 15 yrs (The Netherlands)	4.3 ± 1.1	3.7 ± 1.2
Harris et al. (40) B	~3,700 mg SDA + 2,420 mg/d ALA for 4 mos	*N* = 11 healthy adults (56% female) mean age 38 ± 10 yrs with O3i ≤5% (USA)	4.0 ± 0.9	4.8 ± 1.0^*^
Lemke et al. (41) B	Fortified foods providing 1,600 mg/d SDA for 12 wks	*N* = 50 healthy adults (76% female) mean age 46 (SE 2) yrs (USA)	4.1 (SE 0.2)	4.3 (SE 0.1)^*^
Lemke et al. (42) B	4,200 mg SDA + 1,600 mg/d ALA for 12 wks	*N* = 54 healthy adults (56% female) mean age 49 ± 12 yrs (USA)	4.3 ± 1.1	4.7 ± 0.1^*^
Cao et al. (57) B	3,510 mg ALA + 900 mg/d LA for 8 wks	*N* = 10 healthy adults (60% female) mean age 49 ± 8 yrs (USA)	4.4 (SE 0.3)	4.7 (SE 0.4)
Combe et al. (70) A	Rapeseed oil providing 400 mg EPA + 2,800 mg/d ALA for 6 wks	*N* = 52 older adults (73% female) with history of CVD (France) Female mean age 85 ± 7 yrs Male mean age 83 ± 9 yrs	Men: 4.7 ± 0.9 Women: 4.5 ± 0.8	Men: 4.7 ± 0.8 Women: 4.7 ± 0.9^*^
Combe et al. (70) B	Sunflower oil providing 400 mg EPA + 1,200 mg/d ALA for 6 wks	*N* = 59 older adults (73% female) with history of CVD (France) Female mean age 85 ± 7 yrs Male mean age 83 ± 9 yrs	Men: 4.7 ± 1.0 Women: 4.6 ± 0.8	Men: 4.6 ± 0.9 Women: 4.6 ± 0.7
Ramprasath et al. (71) A	Krill oil providing 600 mg/d mixed n-3 FA for 4 wks	*N* = 24 healthy adults (50% male) mean age 28 ± 5 yrs (Canada)	4.9 ± 1.0	6.0 ± 1.0^*^
Ramprasath et al. (71) B	Fish oil providing 600 mg/d mixed n-3 FA for 4 wks	*N* = 24 healthy adults (50% male) mean age 28 ± 5 yrs (Canada)	5.0 ± 0.8	5.4 ± 1.0^*^
Egert et al. (22) C	Fortified margarine providing 4,400 mg/d ALA for 6 wks	*N* = 24 healthy adults (67% female) mean age 27 ± 6 yrs (Germany)	7.9 ± 2.7	7.1 ± 2.0

Research studies compared different doses, forms of n-3 FA, or composition; thus, each intervention protocol has a separate entry. O3i are presented as means ± SD unless otherwise noted.

* p < 0.05;

# calculated final O3i. ALA, alpha-linolenic acid; LA, linoleic acid; SDA, stearidonic acid; EPA, eicosapentaenoic acid; CVD, cardiovascular disease.

## Discussion

The 58 studies included in this n-3 FA scoping review varied widely in dose, chemical composition, intervention period, and participant characteristics (e.g., age, sex, health status, etc.). However, a common theme that emerged was most participants' baseline O3i ranged from 3 to 5%, well below the recommended 8% for reduced risk of chronic disease. Direct comparison of O3i between studies is challenging due to differences in laboratory assessment. The most successful n-3 FA interventions to clinically and statistically improve O3i supplemented >1,000 mg of combination DHA plus EPA or solely DHA for 12 weeks or longer (Figures 2, 3). Due to limited dietary availability, supplementation and/or fortified foods are necessary for most individuals to reach optimal O3i. Handeland et al. (31) compared n-FA intake from fish vs. supplements, finding that dietary compliance from fish consumption was poor (<40%) and inferior compared to dietary supplements in raising O3i. Fortified foods significantly improved O3i by 1–2% with lower doses than dietary supplements (150–350 mg/d EPA + DHA) (26–30). One exception was the use of fortified margarine, which doubled O3i from 9 to 18% following 6 weeks of 2,300 mg/d DHA in German adults, although these high baseline values and large magnitude of change were not observed in most laboratories (22). Choice of fish consumption, fortified foods, or dietary supplements will depend on baseline O3i, food tolerance, and availability.

Although ALA is the only n-3 FA with a DRI, EPA and DHA are also considered essential for a healthy diet. Furthermore, EPA and DHA improve the O3i to a greater extent than ALA. Since DHA is efficiently incorporated into erythrocyte membranes, DHA improves O3i at lower doses than other n-3 FA. ALA and SDA have limited elongation to EPA and negligible effects on DHA concentrations, which is evident from studies that show minimal effect on O3i. Although EPA elongates to DHA, recent research suggests that DHA rarely retroconverts to EPA, instead slowing turnover of EPA to maintain plasma concentrations (43). On the contrary, DHA undergoes few reactions and is incorporated directly into erythrocyte membranes. As a result, DHA was more effective at raising participants' O3i compared to studies which supplemented equal or greater amounts of EPA.

Metherel et al. (43) completed a secondary review of a study that supplemented 3,256 mg/d DHA for 12 weeks to increase participant O3i by 5% compared to a 3% increase in O3i with an equal dose of EPA (10). Using isotopic carbon tracing to determine the origins of the fatty acids, researchers found EPA supplementation significantly increased concentrations of EPA, DHA, and n-3 docosapentaenoic acid (DPA), a 22-carbon FA synthesized from EPA. DHA supplementation, however, increased concentrations of both DHA and EPA, but lack of carbon tracers in EPA provided evidence of slowed metabolism as opposed to retroconversion. These data suggest that EPA elongates to DHA *via* n-3 DPA as an intermediate, although DHA has limited if any retroconversion to EPA. While EPA may increase O3i to a lesser magnitude than DHA, EPA significantly improves O3i and has important physiological functions such as the synthesis of inflammatory-modulating eicosanoids.

Differences in conversion rates explains why ALA or other forms of n-FA have negligible effects on O3i, which measures the concentration of EPA and DHA in red blood cell membranes. Metherel et al. (43) found that the carbon tracer of the plasma EPA that remained after 12 weeks of DHA supplementation was nearly identical to that of the plasma ALA, suggesting that EPA was synthesized from the preexisting ALA. Therefore, while EPA may be used for *in vivo* synthesis of DHA and n-3 DPA, ALA has little effect on DHA levels and thus, O3i. In summary, n-3 FA's that undergo the fewest interconversions, e.g., DHA and EPA, have the greatest impact on O3i because they are directly incorporated into the membrane.

In addition to dose and length of supplementation, formula is an important consideration. Common dietary supplement formulas include TAG, rTAG, and EE. However, many of the publications included in this review did not specify chemical composition, even though these structures are metabolized differently. Neubronner et al. (36) conducted a clinical comparing 670 mg DHA + 1,010 mg EPA/d as rTAG vs. EE for 6 months. Although O3i significantly improved in both treatment groups, final O3i was greater with rTAG supplementation (13.3 ± 2.2 vs. 12.2 ± 2.3%), supporting greater bioavailability with rTAG than EE. Authors suggested that pancreatic lipases were more efficient at hydrolyzing fatty acids bound to TAG and transporting these fatty acids in the body. Other research supports better bioavailability from TAG, as another study showed significantly greater improvement in O3i by supplementing DHA and EPA acyl-glycerol (a re-esterified polyunsaturated fatty acid) vs. EE for 8 weeks (46). Protocols that specified TAG n-3 FA formulas for ≥10 weeks found 3–7% improvement in O3i, raising the final concentrations to >8% (32, 38, 39, 47). Taken together, these data support suggest that supplemental n-3FA in the form of TAGs or rTAGs are digested and absorbed better than EEs, although, consumption of a fatty meal improves absorption of both forms of n-3 FA.

In addition to dose and forms of n-3 FA, individual characteristics influence O3i response to supplementation. Due to estrogen, women have higher activity of desaturases than men, leading to greater elongation of ALA to EPA and DHA (48). However, advanced age appears to enhance response to dietary EPA and DHA in both men and post-menopausal women, perhaps owing to reduced turnover of n-3 FA (49). While these factors are important, they account for a small percentage of variability in response to n-3 FA intake. Flock et al. (50) estimated that body weight, baseline O3i, age, physical activity, and sex together accounted for 10% of response, compared to dose, which alone explained 68%. Incorporating this study along with 13 other intervention trials, Walker et al. (51) estimated that 62% of variance in response was explained by EPA and DHA dose, baseline O3i, and formula of supplement, eliminating age and sex from the final model. Neither of these analyses accounted for genotype, which was found to explain 24% of O3i in a model from data collected in the Framingham Heart Study (49). Across the studies included in this review, analyses that separated sex and age did not find large differences in response to supplementation, although some noted baseline O3i were higher in females (13, 50).

Baseline O3i and whole body kinetics further affect response to dietary n-3 FA intake. Individuals with lower n-3 FA concentrations may better incorporate EPA and DHA into erythrocyte membranes, potentially reaching a saturation point (50). Research suggests that erythrocyte n-3 FA concentrations respond in a dose-dependent manner until reaching equilibrium, typically 4–6 months from the start of intervention (52). Studies included in this review that assessed midpoint O3i values showed mixed results, with one showing a plateau after only 6 weeks (53), and others after 3 months (33, 36, 37). One study, however, found continued O3i increases through 4 months of supplementation (23), while another found small decreases from 6 months to 1 year (16). This research suggests that lower doses are likely necessary to maintain O3i than to increase levels from a low baseline.

Limitations to this review include a lack of standardized procedure for assessment of O3i, contributing to variable baseline concentrations. Full analysis of laboratories procedures is beyond the scope of this review.

## Conclusion

The O3i was first validated as a risk factor for cardiovascular disease by Harris and Schacky (7) in 2004 and remains a generally accepted measure for determining omega-3 status. The majority of the world population has O3i levels well below the recommended target of ≥8%. Across the 58 studies reviewed, only three study samples from Germany and Austria began with a mean baseline assessment ≥8% (22–24), and laboratory assessment methods may have accounted for these high baseline values. Most mean baseline O3i ranged from 3 to 6%, suggesting that most individuals would benefit from improved omega-3 status, leaving the question of how best to reach optimal levels.

Few individuals meet recommendations for fish consumption, which appear insufficient to reach optimal O3i status even for those who do consume two servings per week (1). The sole study protocol that provided fish for 3 meals per week found low compliance in Norwegian teenagers, although adults may be more tolerant. Dietary interventions with fortified foods showed promise in low doses, highlighting an area for future research. Most supplementation studies did not detail the diet of their participants, using general descriptions such as “low or no habitual consumption of oily fish” (50) or no description at all. Since diet, n-3 FA dose and chemical composition influence individual outcomes, Walker et al. (51) developed a model to predict O3i response to 16 weeks of supplementation. With a baseline O3i of 2–4%, an individual would need to consume 1,500–2,250 mg/d EPA and DHA as TAG's or 2,250–3,250 mg/d EE's to reach 8%. For individuals with baseline O3i from 4 to 6%, a dose of 1,000–1,500 mg/d as TAG's or 1,500–2,250 mg/day as EE's is recommended.

## Practical applications

Few individuals meet the target O3i of 8%, increasing their risk of cardiovascular disease among other chronic ailments. Improving O3i with fish alone is neither realistic nor sustainable, leaving fortified foods and/or third-party tested dietary supplements to close the gap. Generally, the lowest doses shown to be effective in raising O3i to recommended levels were >1,000 mg of combination DHA plus EPA or solely DHA for 12 weeks or longer. Supplements composed of TAG were more bioavailable and thus more effective than other formulas. Based on the data evaluated, practical recommendations to improve O3i to ≥8% are consumption of 1,000–1,500 mg/day EPA plus DHA as TAG for at least 12 weeks to improve levels. Those with baseline O3i <4% will likely need longer time periods of supplementation and/or higher doses. Research evaluated was to improve O3i rather than maintain healthy levels so the appropriate dose for maintenance is an area for future research.

## Data availability statement

The original contributions presented in the study are included in the article/supplementary material, further inquiries can be directed to the corresponding author.

## Author contributions

MD, MR, and LW contributed to design and editing of the manuscript. MD conducted the literature review with assistance by LW and MR. MD and LW drafted the manuscript. MR critically edited the manuscript. All authors read and approved the final submission.
